# MiR-181a-5p Regulates NIS Expression in Papillary Thyroid Carcinoma

**DOI:** 10.3390/ijms22116067

**Published:** 2021-06-04

**Authors:** Wojciech Gierlikowski, Katarzyna Broniarek, Łukasz Cheda, Zbigniew Rogulski, Marta Kotlarek-Łysakowska

**Affiliations:** 1 Department of Internal Medicine and Endocrinology, Medical University of Warsaw, Banacha 1a, 02-097 Warsaw, Poland; 2 Faculty of Medicine, Medical University of Warsaw, Żwirki i Wigury 61, 02-091 Warsaw, Poland; broniarekkatarzyna@gmail.com; 3 Faculty of Chemistry, Biological and Chemical Research Centre, University of Warsaw, Żwirki i Wigury 101, 02-089 Warsaw, Poland; lcheda@chem.uw.edu.pl (Ł.C.); rogul@chem.uw.edu.pl (Z.R.); 4 Warsaw Genomics, Żwirki i Wigury 101, 02-089 Warsaw, Poland; marta.kotlarek-lysakowska@warsawgenomics.pl

**Keywords:** NIS, *SLC5A5*, microRNA, miR-181a-5p, papillary thyroid carcinoma, thyroid carcinoma, PTC

## Abstract

NIS is a potent iodide transporter encoded by the *SLC5A5* gene. Its expression is reduced in papillary thyroid carcinoma (PTC). In this study we analyzed the impact of miR-181a-5p on NIS expression in the context of PTC. We used real-time PCR to analyze the expression of *SLC5A5* and miR-181a-5p in 49 PTC/normal tissue pairs. Luciferase assays and mutagenesis were performed to confirm direct binding of miR-181a-5p to the 3′UTR of *SLC5A5* and identify the binding site. The impact of modulation of miR-181a-5p using appropriate plasmids on endogenous NIS and radioactive iodine accumulation was verified. We confirmed downregulation of *SLC5A5* and concomitant upregulation of miR-181a-5p in PTC. Broadly used algorithms did not predict the binding site of miR-181a-5p in 3′UTR of *SLC5A5*, but we identified and confirmed the binding site through mutagenesis using luciferase assays. In MCF7 and HEK293-flhNIS cell lines, transfection with mir-181a-expressing plasmid decreased endogenous *SLC5A5*, whereas silencing of miR-181a-5p increased it. We observed similar tendencies in protein expression and radioactive iodine accumulation. This study shows for the first time that miR-181a-5p directly regulates *SLC5A5* expression in the context of PTC and may decrease efficacy of radioiodine treatment. Accordingly, miR-181a-5p may serve as an emerging target to enhance the efficacy of radioactive iodine therapy.

## 1. Introduction

The ability of the thyroid gland to accumulate iodide has been known since the end of the 19th century [1]. Diagnostic use of radioiodine dates back to the 1930s [2], and since the 1940s radioiodine has been used for the treatment of thyroid carcinoma [3]. The capability of thyroid to collect iodide, necessary for fulfilling its physiological function, which is synthesis of iodine-containing thyroxin and triiodothyronine [4], results from the activity of mainly one protein—sodium/iodide symporter (NIS). NIS is a multi-pass membrane glycoprotein located on the basolateral membrane of thyroid follicular epithelial cells, which is able to concentrate iodine, creating an up to 40-fold gradient [5]. It consists of 643 amino acids and is encoded by the *SLC5A5* gene [6]. Soon after its molecular characterization, it was found that expression of NIS is reduced in thyroid tumors compared to normal thyroid tissue [7].

Thyroid carcinoma is the most common endocrine malignancy, and its incidence is rising. Papillary thyroid carcinoma (PTC) comprises about 85% of the cases [8]. Genetics of PTC was characterized as a part of The Cancer Genome Atlas (TCGA) project, revealing generally low somatic mutation density, with mainly MAPK-related genes (i.e., BRAF, NRAS, HRAS and KRAS) [9]. The presence of the BRAF^V600E^ mutation, occurring in approximately 50% of PTC [10], leads to NIS repression by TGFβ pathway [11,12], decreases its expression by promotor deacetylation [13] and disrupts its trafficking to cell membranes [14].

MicroRNAs (miRNAs, miRs) are short (19–25 nucleotides) non-coding RNA molecules that usually function as inhibitors of the expression of protein-coding genes. Previous reports suggest that microRNAs altogether regulate around 60% of the human genome [15]. Mature microRNAs bind to complementary sequences in mRNA 3′untranslated regions (3′UTRs), thus inhibiting translation or promoting mRNA degradation [16]. Many cancers, including PTC, exhibit aberrant expression of microRNAs [17], which leads to deregulation of expression of numerous protein-coding genes.

Changes in microRNA expression patterns are typical for different pathological conditions and can be used to diagnose thyroid lesions (as revised in [18]). Aberrant expression of microRNAs leads to altered expression of their target genes, contributing to promotion and progression of cancer. Since the expression of microRNAs can be modulated, this phenomenon can be further used for the tailoring of adjuvant cancer therapies [19].

So far, it has been reported that NIS is regulated by miR-146a-3p [20], miR-146b-3p [21,22], miR-339-5p [23] and miR-875-5p [24].

During our previous work, we noticed that miR-181a-5p targets the apical iodine transporter (AIT, encoded by *SLC5A8*) [25] and was predicted to regulate pendrin by in silico analyses. Consequently, we expected that overexpression of miR-181a5p would have led to increased radioiodine retention, whereas silencing, to decreased radioiodine retention. As the results were contrary to expected, we hypothesized that miR-181a-5p regulates NIS as well and performed the present study, aiming to test this hypothesis.

## 2. Results

### 2.1. The Expression of SLC5A5 Is Lowered in PTC

The samples consisted of two groups: cancer tissue (*n* = 49, PTC-T) and control tissue (paired normal tissue from the same patient; *n* = 49, PTC-N). The patients represented all stages of the disease excluding IVB; 44 (90%) tumors represented the classic variant of PTC (PTCcv) and 5 (10%) were the follicular variant of PTC (PTCfv). The medium tumor diameter was 16.23 mm (range: 1–73 mm). Patient characteristics are summarized in Table 1. BRAF^V600E^ mutation status was checked as described previously [17] in 41/49 tumors, identifying the mutation in 18 of them.

**Figure 1:**
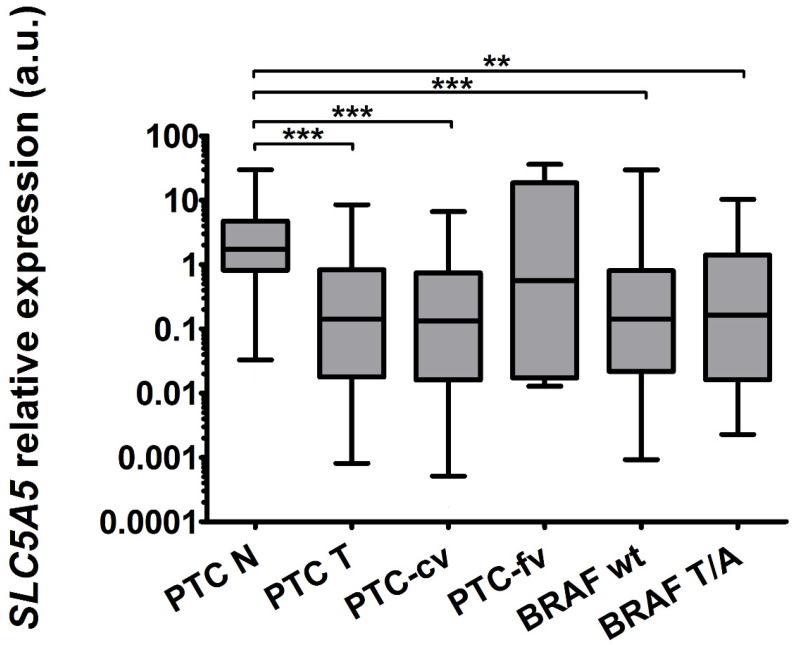
The expression of *SLC5A5* was 12.27-fold (*p* < 0.0001) reduced in PTC (PTC-T, *n* = 49) compared with normal adjacent tissue (PTC-N, *n* = 49). The reduction was more profound in the classic variant of PTC (PTC-cv, *n* = 44, 13.16-fold, *p* < 0.0001) compared with the follicular variant (PTC-fv, *n* = 5, 3.11-fold, non-significant). The reduction was observed both in the BRAF wild-type group (BRAF wt, *n* = 23, 12.27-fold, *p* = 0.0009) and BRAF mutated group (BRAF T/A, *n* = 18, 10.65-fold, *p* = 0.007; please note that expression in tumors was compared with expression in matched controls only). The graph shows the expression of *SLC5A5* in thyroid tissue samples normalized against *HPRT*. Data are expressed as median, interquartile range and 5–95 percentile. Statistical analysis was performed with a Mann–Whitney U test (** *p* < 0.01, *** *p* < 0.001). Logarithmic scale was used.

In 43/49 (87%) tissue pairs, the expression of *SLC5A5* was lower in tumor than in control tissue. The median decrease in the whole group was 12.27-fold (*p* < 0.0001). The difference was significant regardless of BRAF^V600E^ status (T, wild type, *n* = 23 and mutated, T/A, *n* = 18, Figure 1). There was no apparent correlation between the expression of *SLC5A5* and histopathological features.

### 2.2. The Expression of miR-181a-5p Is Upregulated in Tumor Tissue

**Figure 2:**
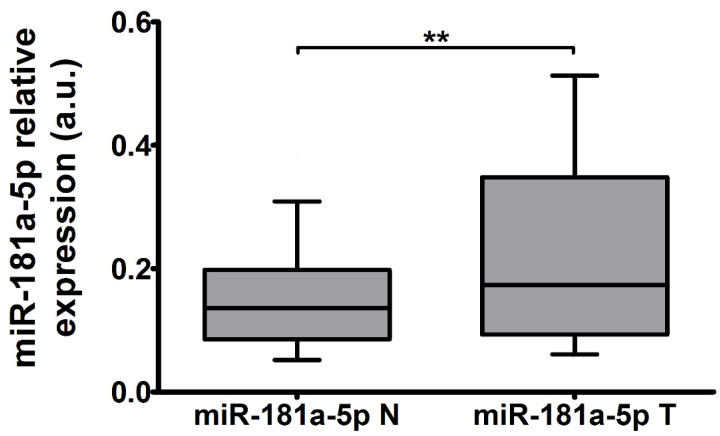
The expression of miR-181a-5p normalized to U44 in PTC-T versus PTC-N tissue (*n* = 49). The expression was increased by 27% (*p* = 0.0015). Data are expressed as median and 10–90 percentile. Statistical analysis was performed with a Wilcoxon *t*-test to compare expression of miRNA in PTC-T vs. PTC-N tissue, ** *p* < 0.01.

The expression of miR-181a-5p in the studied thyroid tissue pairs was increased by 27% (*p* = 0.0015, *n* = 49, Figure 2). No correlation between *SLC5A5* and miR-181a-5p expression was found.

### 2.3. Genes Expression in TCGA Cohort

**Figure 3:**
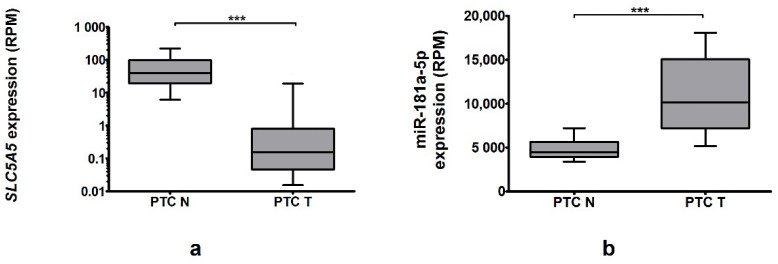
In TCGA cohort (**a**) *SLC5A5* expression was decreased in tumor 13.7-fold (*p* < 0.001), whereas (**b**) miR-181a expression was increased 2.27-fold (*p* < 0.001). Data are expressed as median and 10–90 percentile. Logarithmic scale was used. Statistical analysis was performed with a Wilcoxon *t*-test to compare expression of miRNA in PTC-T vs. PTC-N tissue, *** *p* < 0.001.

In the TCGA cohort (*n* = 59) we confirmed *SLC5A5* downregulation, with a decrease of 13.7-fold (*p* < 0.0001, Figure 3a). This change was accompanied by an increase in miR-181a-5p expression (2.27-fold, *p* < 0.0001, Figure 3b). There was weak, negative correlation between the expression of these particles (Spearman’s r -0.39, *p* = 0.002).

### 2.4. Investigation of Binding of miR-181a-5p with 3′UTR of SLC5A5

#### 2.4.1. Analysis Employing Broadly Used Algorithms Does Not Reveal Binding of miR-181a-5p with 3′UTR of SLC5A5

None of the used algorithms (i.e., miRanda, TargetRank, mirdb.org, PicTar and DIANA microT-CDS) predicted *SLC5A5* 3′UTR as a target for miR-181a-5p.

#### 2.4.2. Identification and Confirmation of miR-181a-5p Binding Site

**Figure 4:**
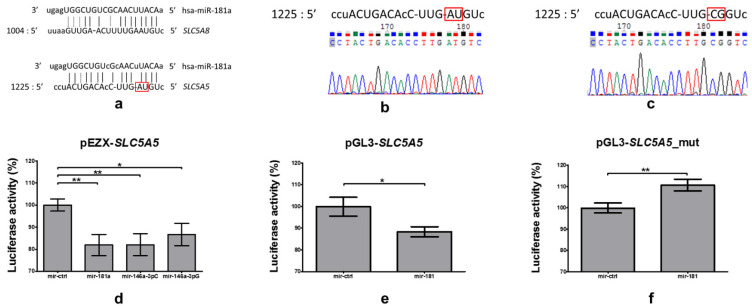
(**a**) In silico analysis revealed similarity between region of 3′UTR of *SLC5A8*, which binds with miR-181a-5p (upper part), and region of putative binding of miR-181a-5p in 3′UTR of *SLC5A5* (lower part; adapted from microrna.org). (**b**) A 118bp fragment of 3′UTR of *SLC5A5*, containing a putative binding site for miR-181a-5p, was subcloned into pGL3-MCS_down_luc plasmid. (**c**) Two nucleotides (1241–1242) were mutated in putative binding site. (**d**) Co-transfection of HeLa cells with pEZX-*SLC5A5* and plasmids expressing mir-181a, mir-146a-3pC or mir-146a-3pG resulted in a reduction in luciferase activity by 18 (*p* = 0.005), 18 (*p* = 0.006) and 13% (*p* = 0.033), respectively, in comparison with mir-ctrl. (**e**) Co-transfection of HeLa cells with pGL3-*SLC5A5*, containing a 118bp fragment of 3′UTR of *SLC5A5* with putative miR-181a-5p binding site, and plasmids expressing mir-181a, led to a 12% (*p* = 0.032) decrease in luciferase activity. (**f**) Co-transfection of HeLa cells with pGL3-*SLC5A5*_mut, containing a 118bp fragment of 3′UTR of *SLC5A5* with mutations in putative miR-181a-5p binding site, and plasmids expressing mir-181a, did not lead to a reduction in luciferase activity—an 11% (*p* = 0.0085) increase was observed. Luciferase activity is shown as a percentage relative to the control. The results are normalized by *Renilla* luciferase and derived from three independent experiments, each performed in triplicates. The graph shows the mean, along with deviations from mean (SEM). Statistical analysis was performed using an unpaired *t*-test (* *p* < 0.05, ** *p* < 0.01).

We analyzed the 3′UTR of *SLC5A8* in the region predicted to interact with miR-181a-5p (Figure 4a, upper part) and found a similar sequence in the 3′UTR of *SLC5A5*, between nucleotides 1226 and 1243 (Figure 4a, lower part). We subcloned a 118bp fragment of *SLC5A5* 3′UTR containing the identified sequence (i.e., nucleotides 1152–1269) into pGL3-MCS_down_luc plasmid [26] (pGL3-*SLC5A5*, as shown in Figure 4b) and mutated two nucleotides (i.e., nucleotides 1241–1242) in the putative binding site (pGL3-*SLC5A5*_mut, Figure 4c).

Co-transfection with pEZX-*SLC5A5* and mir-181a decreased luciferase activity by 18% (*p* = 0.005, Figure 4d). A similar effect was observed upon transfection with plasmids expressing two isoforms of mir-146a serving as a positive control, namely mir-146a-3pC or mir-146a-3pG, which led to reduction by 18 (*p* = 0.006) and 13% (*p* = 0.033, Figure 4d), respectively.

When a 118bp fragment of *SLC5A5* 3′UTR, containing the putative binding site of miR-181a-5p, was subcloned into a pGL3-*SLC5A5* construct, a reduction in luciferase activity was preserved, with a decrease by 12% (*p* = 0.032, Figure 4e). Upon introduction of a mutation in the pGL3-*SLC5A5*_mut construct, the ability of binding was lost—we observed an unspecific increase in luciferase activity by 11% (*p* = 0.0085, Figure 4f).

### 2.5. miR-181a-5p Affects Expression of Endogenous SLC5A5

#### 2.5.1. Plasmid Functionality Verification

**Figure 5:**
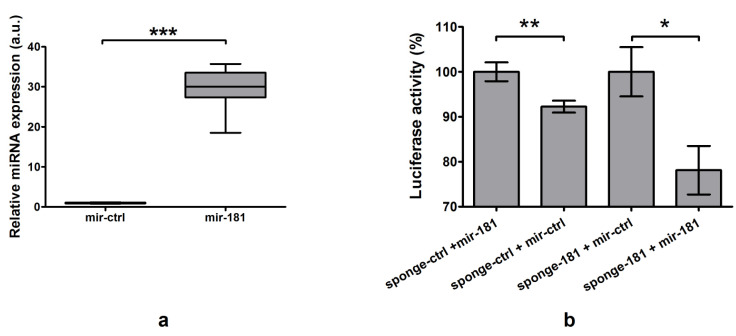
Functionality of obtained plasmids. (**a**) Transfection of HeLa cells with mir-181-expressing plasmid led to an increase in expression of miR-181a-5p measured in real-time PCR by 29-fold (*p* < 0.0001). (**b**) Co-transfection of HeLa cells with sponge-ctrl plasmid and mir-ctrl led to a decrease in luminescence activity by 8% (*p* = 0.004), when compared with co-transfection with sponge-ctrl and mir-181-expressing plasmids. Similarly, co-transfection with sponge-181 and mir-181 decreased luciferase activity by 22% (*p* = 0.012), when compared with co-transfection with sponge-181 and mir-ctrl. Data are expressed as mean values +/− SEM. Statistical analysis was performed using an unpaired *t*-test (* *p* < 0.05, ** *p* < 0.01, *** *p* < 0.001).

Functionality of the mir-expressing plasmid was confirmed in HeLa cells using TaqMan kits. Transfection with mir-181-expressing plasmid led to a 29-fold increase in its expression (*p* < 0.0001, Figure 5a). Functionality of sponge-expressing plasmids was verified by co-transfecting HeLa cells with sponge- and microRNA-expressing plasmid with appropriate controls. Co-transfection with an appropriate microRNA-expressing plasmid (i.e., expected to bind to the expressed particle) led to reduction in luciferase activity when compared with control transfections—by 8% in the case of control plasmids (*p* = 0.004), and by 22% for sponge-181 and mir-181 co-transfection (*p* = 0.012; Figure 5b).

#### 2.5.2. Impact of Overexpression and Silencing of miR-181a-5p on NIS Expression and Function

Total RNA isolated from MCF7 cells was submitted to whole transcriptome sequencing, but no significant results were found in the case of both overexpression and silencing of miR-181a-5p.

**Figure 6:**
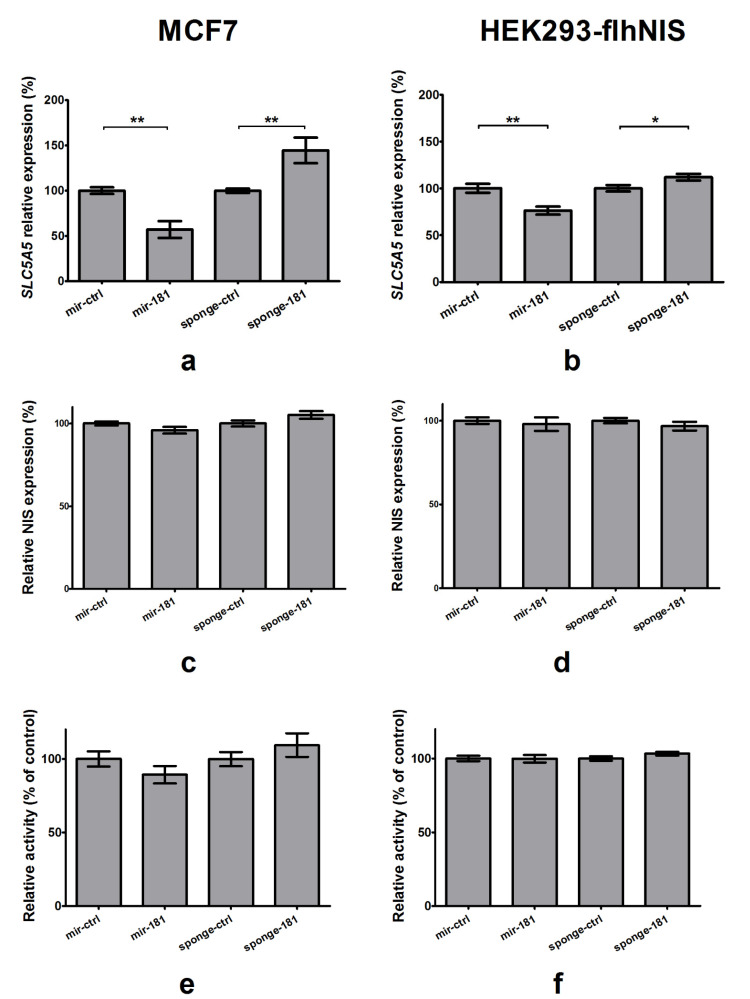
Impact of miR-181a-5p on NIS expression and function. (**a**) Transfection of MCF7 cells with mir-181-expressing plasmid reduced *SLC5A5* mRNA by 43% (*p* = 0.001), whereas silencing of miR-181a-5p using sponge decreased *SLC5A5* by 45% (*p* = 0.0045). (**b**) In HEK293-flhNIS, overexpression of mir-181 led to a reduction in *SLC5A5* mRNA expression by 24% (*p* = 0.0045), while miR-181a-5p silencing increased *SLC5A5* expression by 12% (*p* = 0.036). NIS expression measured using ELISA in (**c**) MCF7 cells induced with all-trans retinoic acid and dexamethasone, and (**d**) HEK293-flhNIS cells. Tendencies for lower expression upon transfection with mir-181-expressing plasmid are visible for both cell lines. Tendency for higher expression can be seen upon transfection with a plasmid silencing miR-181a-5p in MCF7 cell line. Radioiodine uptake in (**e**) MCF7 cells induced with all-trans retinoic acid and dexamethasone, and (**f**) HEK293-flhNIS cells. Tendencies for lower retention upon transfection with mir-181-expressing plasmid and for higher expression upon transfection with a plasmid silencing miR-181a-5p are visible for both cell lines. Data are expressed as mean values +/− SEM. Statistical analysis was performed using a Mann–Whitney test (**a**,**b**) and an unpaired *t*-test (**c**–**f**); * *p* < 0.05, ** *p* < 0.01.

Transfection of the MCF7 cell line upon induction with ATRA and dexamethasone with plasmids expressing mir-181a resulted in a reduction in *SLC5A5* mRNA by 43% (*p* = 0.001), whereas suppression of miR-181a-5p using an appropriate sponge increased *SLC5A5* expression by 45% (*p* = 0.0045; Figure 6a). When HEK293-flhNIS cells were transfected with plasmids expressing mir-181a, *SLC5A5* mRNA was decreased by 24% (*p* = 0.0045), whereas silencing of miR-181a-5p using an appropriate sponge led to the induction of *SLC5A5* expression by 12% (*p* = 0.036; Figure 6b).

On the protein level, we observed similar, but not statistically significant, tendencies in MCF7 cells (Figure 6c), and a tendency for NIS suppression in HEK293-flhNIS cells upon transfection with mir-181a (Figure 6d). Additionally, in radioactive iodine uptake tests in MCF7 cells the tendencies were clear (Figure 6e), whereas in HEK293-flhNIS cells they were barely visible (Figure 6f).

**Figure 7:**
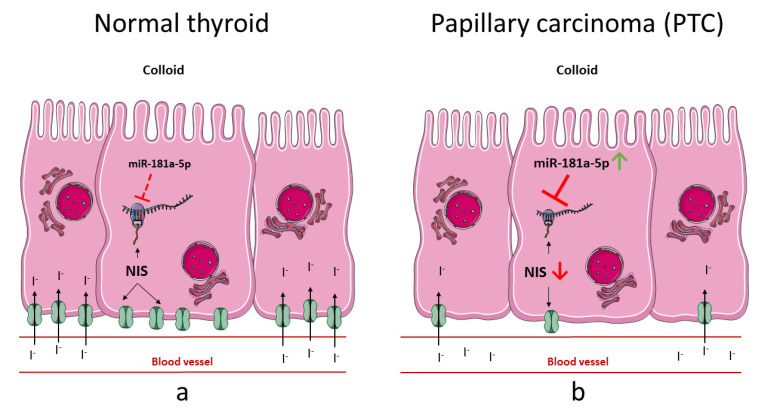
Graphical summary of the study. MicroRNA miR-181a-5p regulates NIS, (**a**) leading to fine-tuning of NIS in normal thyroid tissue, and (**b**) contributing to severe NIS downregulation in the context of PTC. Figure contains elements available at Servier Medical Art repository, licensed under a Creative Commons Attribution 3.0 Unported License. http://smart.servier.com/ accessed 28 March 2021.

A graphical summary of the study is shown in Figure 7.

## 3. Discussion

In this study, we show, for the first time, that miR-181a-5p directly regulates expression of *SLC5A5*. Our study adds a new part of the puzzle, after reports regarding miR-146a-3p, miR-146b-3p, miR-339-5p and miR-875-5p.

NIS, a protein encoded by the *SLC5A5* gene, is crucial for thyroid physiology, and in the case of differentiated thyroid carcinomas, such as PTC, is essential to enable radioiodine therapy, which is an important part of adjuvant PTC treatment [27]. Numerous previous studies, including TCGA, provide data regarding the reduction in *SLC5A5* expression in PTC (some of the studies were revised recently [28]). Our results confirm these findings. On the contrary to previous reports, in our cohort *SLC5A5* expression in tumor did not depend on BRAF^V600E^ status, but this may result from population differences. It should be also emphasized that NIS function may be impaired by BRAF^V600E^ mutation not only through downregulation, but also by impaired trafficking to the cell membrane [29]. BRAF^V600E^ mutation was proposed as a predictor of radioiodine therapy effectiveness in PTC [30]; however, data undermining this approach also exist [31,32]. What is more, clinical studies which tested the ability of MAPK pathway inhibitors, including BRAF inhibitors, resulted in rather disenchanting effects (as revised in [33]). Thus, it seems that the presence of a BRAF^V600E^ mutation is not a sufficient condition for resistance to radioiodine therapy.

The role of microRNA-mediated gene regulation is a growing matter of interest in thyroid carcinomas. As mentioned, so far regulation of NIS by miR-146a-3p, miR-146b-3p, miR-339-5p and miR-875-5p was shown. Our previous, unpublished data draw our attention to miR-181a-5p as another putative suppressor of NIS. Data regarding miR-181a-5p in PTC is not vast; however, recent analysis underlined its putatively important role as a factor contributing to dysregulation of numerous genes [34]. In our cohort, miR-181a-5p was overexpressed in PTC, which stands in line with previous findings [17,35]. We did not observe higher levels of miR-181a-5p in the case of BRAF^V600E^ mutation, similarly to a recent Czech study [36], but on the contrary to a previous study in the Chinese population [37]. We also did not find a negative correlation between *SLC5A5* and miR-181a-5p, but we noted such a correlation in the TCGA cohort. Lack of a negative correlation in our cohort may mirror the complexity of regulation of *SLC5A5* expression in PTC. It is worth emphasizing that dividing PTC cases into BRAF-like and RAS-like does not exhaust the complexity of its genetics [9,38].

As the broadly used algorithms [39,40] did not predict interaction between miR-181a-5p and *SLC5A5* 3′UTR, we further investigated *SLC5A5* 3′UTR, among others, scanning for similarity with *SLC5A8* 3′UTR, which we have shown to interact with miR-181a-5p previously [25]. Indeed, we found a similar sequence down 1225 nt of the 3′UTR of *SLC5A5*. Using a luciferase assay, we confirmed that the product from the mir-181 precursor interacts with the *SLC5A5* 3′UTR, as was previously shown for mir-146a (for its two alleles, differing by a single nucleotide polymorphism [20,26]). Interaction was maintained when a 118 nt fragment, containing an identified region, was subcloned into another plasmid, but was abolished upon introducing mutations at the predicted binding site. The binding site is not a canonical one, with at least six consecutive, complementary nucleotides [41], nor a non-canonical type, with a bulge within 3′UTR [42]—in our case the expected bulge occurs in a microRNA strand. Perfect pairing may be observed, among others, in the case of nucleotides 2–4 of miR-181a-5p, which is essential for the initial step of microRNA interaction with targeted mRNA [43], whereas the complementarity of nucleotides 4–5 allows for strong binding [44]. A bulge nucleotide within microRNA does not exclude the interaction; what is more, pairing at the 3′ end of microRNAs (especially involving nucleotides 13–16, as in this case) can compensate for seed mismatches [15]. Experimental identification of the microRNA binding site (accordingly to [45]), which was not predicted by broadly used algorithms, is one of the main strengths of the study.

We showed that overexpression of miR-181a-5p (using a mir-expressing plasmid) led to downregulation of *SLC5A5* mRNA in both MCF7 and HEK293-flhNIS cell lines, whereas silencing of miR-181a-5p resulted in its upregulation. We checked for whole-transcriptome effects using NGS, but the results speak against serious deregulation of numerous genes at the whole transcriptome level. Tendencies similar to changes observed at the RNA level were shown at the level of protein expression and cell function, measured as radioiodine uptake. Dysregulation at the protein level was not more prominent than at the RNA level, which is in agreement with the findings that in mammals mRNA destabilization is the main effect of microRNA action [46], thus mRNA changes are believed to provide a nearly quantitative readout of the miRNA-mediated repression [47]. It should be emphasized that a decrease in radioiodine accumulation may be partially compromised by a simultaneous decrease in radioiodine efflux, as miR-181a-5p regulates *SLC5A8* [25], encoding the apical iodine transporter, and is predicted (by miRanda, TargetRank, and DIANA microT-CDS) to target *SLC26A4*, encoding pendrin, both of which probably take part in mediating the efflux [48]. This stands in line with the concept that a single microRNA may regulate different genes involved in a single process through canonical and non-canonical interactions [49]. The use of MCF7 and HEK293-flhNIS cell lines instead of the thyroid cancer-derived cell line may be seen as a limitation; however, human thyroid cancer cell lines exhibit barely detectable levels of NIS, similarly to the tumor tissue, making such a study hard to perform. Importantly, both cell lines used in the study are accepted models and were previously used for a similar purpose [20,23]. Since the principle of action of microRNAs on the target gene does not depend on cell type, the finding that miR-181a-5p directly reduces *SLC5A5* expression in MCF7 and HEK293-flhNIS cells could be extended to suggest that overexpression of miR-181a-5p at least in part accounts for the reduced expression of *SLC5A5* in PTC.

To conclude, reduced expression of NIS in PTC seems to result, among other factors, from upregulation of miR-181a-5p. This phenomenon may contribute to resistance to radioactive iodine therapy, and the significance of miR-181a-5p as predictor of treatment efficiency remains to be elucidated. Since microRNA levels can be modulated, miR-181a-5p should be considered a promising therapeutic target.

## 4. Materials and Methods

### 4.1. Tissue Samples

Tissue samples were obtained with the permission of the Bioethics Committee of the Medical University of Warsaw (no. KB/184/2009) from patients with papillary thyroid carcinoma and collected at Genomic Medicine, Medical University of Warsaw. Each patient signed informed consent prior to surgery. The samples consisted of two groups: cancer tissue (*n* = 49, PTC-T) and control tissue (paired normal tissue from the same patient; *n* = 49, PTC-N). PTC was diagnosed by histology according to World Health Organization 2004 criteria. The patients represented all stages of the disease excluding IVB; 44 (90%) tumors represented the classic variant of PTC (PTCcv) and 5 (10%) were the follicular variant of PTC (PTCfv). The medium tumor diameter was 16.23 mm (range: 1–73 mm). Patient characteristics are summarized in Table 1. BRAF^V600E^ mutation status was checked as described previously [17] in 41/49 tumors, identifying the mutation in 18 of them.

### 4.2. Real-Time PCR

Total RNA was extracted with a standard TRIzol–chlorophorm method (RNA Extracol, EURx, Gdańsk, Poland). Quality and concentration of RNA was assessed using a NanoDrop2000 spectrophotometer (Thermo Scientific, Waltham, MA, USA); A260/280 and A260/230 ratios between 1.8 and 2.2 were considered as satisfactory. To establish *SLC5A5* mRNA level, RNA was reverse transcribed using M-MLV reverse transcriptase (Promega, Madison, WI, USA), and gene expression was analyzed in a real-time SQ-PCR assay using a Light Cycler 480 (Roche, Basel, Switzerland). *HPRT* served as an internal control. Primers are listed in Table S1. Reverse transcription and real-time PCR of miR-181a-5p was performed using a specific TaqMan probe with U44 as an internal control (Life Technologies, Waltham, MA, USA, cat. no. 000480 and 001094, respectively). Relative quantification of expressed RNA was calculated using the standard 2^−ΔCt^ method.

### 4.3. Comparison of the Results with the TCGA Data

The Cancer Genome Atlas data was accessed 11 February 2016 using the tcga-data.nci.nih.gov portal. We identified 59 cases for whom the mRNA-Seq and miRNA-Seq level 3 data for tumor and healthy control tissue were available. Expression of *SLC5A5*, miR-181a-1-5p and miR-181a-2-5p were normalized as reads per million (RPM). The obtained normalized data was used for the analysis of expression and correlation between the *SLC5A5* and miRNA. Subgroups of PTCcv vs. PTCfv and BRAF wild-type vs. V600E-mutated were analyzed. Since the miR-181a-1 and miR-181a-2 isoforms are undistinguishable in the TaqMan analysis, both isoforms were counted together, according to the conception of “seed power”, i.e., the conception that all the miRNAs regulate common target genes [50].

### 4.4. In Silico Identification of Binding Site of miR-181a-5p in SLC5A5 3′UTR

Sequence of *SLC5A5* 3′UTR (1297nt) was obtained from Ensembl Database (www.ensembl.org, accessed on 2 May 2014). MicroRNAs potentially binding *SLC5A5* 3′UTR were predicted using miRanda algorithm (microrna.org), TargetRank (http://genes.mit.edu/targetrank/), mirdb.org, PicTar (https://pictar.mdc-berlin.de/) and DIANA microT-CDS (http://diana.imis.athena-innovation.gr/DianaTools/index.php?r=MicroT_CDS, all accessed on 19 October 2018). Clone Manager [51] was used for analyzing *SLC5A5* 3′UTR, including comparison with different targets of miR-181a-5p, planning cloning of its fragment and planning mutagenesis.

### 4.5. MicroRNA Cloning and MicroRNA-Sponges Preparation

The influence of miRNA-181a-5p on endogenous *SLC5A5* levels was analyzed using microRNA- and microRNA-sponge-expressing plasmids. The sequence encoding the precursor (referred to as mir) for miR-181a-5p was identified based on the Ensembl Database (www.ensembl.org, accessed on 2 May 2014). Primer pairs (Table S1) were created using Clone Manager [51], and restriction sites for *Hind*III (forward primer) and *Eco*RV (reverse primer) were added. Formation of hairpin structure by the product was confirmed with RNA Shapes software (https://bibiserv2.cebitec.uni-bielefeld.de/rnashapes, accessed on 2 May 2014). Precursors were amplified on the template of DNA isolated from leukocytes of healthy donors using Opti Taq 2x PCR Master Mix (EURx). The product and the pcDNA3 plasmid (Life Technologies) were digested with the *Hind*III and *Eco*RV restriction enzymes (Promega) and ligated using T4 ligase (Promega). As the cloned sequence contained microRNA precursor, the plasmid is referred to as “mir-expressing” [52]. A control sequence (Table S1), expected to be transcribed and processed like microRNAs, but not targeting any 3′UTRs, was cloned similarly.

A tandem sequence complementary to the binding site of miR-181a-5p was synthetized, amplified using primers containing restriction sites for *Xho*I and *Pst*I (Table S1) and cloned into pGL3-MCS_down_luc, pGL3 plasmid with multicloning site moved downstream of luciferase gene [26], resulting in microRNA sponges [53]. Its specificity was confirmed using MirTarget algorithm (www.mirdb.org, accessed on 10 February 2019). A control sequence, expected to bind with barely any microRNAs, was synthetized and cloned similarly (Table S1).

All constructs were sequenced using Sanger technique. Overexpression upon transfection with expressing plasmid was confirmed in HeLa cells using a TaqMan kit. MicroRNA sponge was validated in HeLa cells co-transfected with the sponge and mir-181a-expressing plasmid. HeLa cells (obtained from ATCC^®^, passage 9th to 12th) were seeded on 12-well plates, 2 × 10^5^ cells per well, and after 24 h transfected in combinations with 500 ng of mir-181 or mir-ctrl and 100 ng of sponge-181a or sponge-ctrl. As a control, 500 ng of pRL-TK plasmid (Promega), expressing *Renilla* luciferase was used. Then, 48 h after transfection, cells were subjected to luciferase assay (Promega), using GloMax-Multi Detection System (Promega) according to the manufacturer’s protocol.

### 4.6. Analysis of miR-181a-5p-Mediated Regulation of SLC5A5

Direct binding of miR-181a-5p to *SLC5A5* was analyzed using luciferase assay. HeLa cells (obtained from ATCC^®^, passage 9th to 12th) were transfected with the pEZX-*SLC5A5* reporter vector (GeneCopoeia, Rockville, MD, USA, cat. no. HmiT017390-MT01) containing the 3′UTR of *SLC5A5* cloned downstream of the coding sequence of the firefly luciferase, and with mir-expressing plasmids. Plasmids expressing miR-146a-3pC and miR-146a-3pG served as a positive control [20]. Then, 48 h after pEZX-*SLC5A5* transfection, cells were subjected to luciferase assay (Promega), using GloMax-Multi Detection System (Promega) according to the manufacturer’s protocol. Constitutively expressed *Renilla* luciferase served as the internal control. Subsequently, binding region in *SLC5A5* 3′UTR was identified by subcloning and mutagenesis, as described in the Results.

To analyze the effect of miR-181a on endogenous *SLC5A5* expression, appropriate mir- or sponge-expressing plasmids were used. MCF-7 cells (ATCC^®^ HTB-22^TM^) were seeded in medium containing 45% DMEM, 45% F-12 and 10% FBS with addition of 1 µM of all-trans-retinoic acid and dexamethasone (compounds inducing NIS expression [54,55]) onto 12-well plates using 2 × 10^5^ cells per well. HEK293-flhNIS, stably transfected with full-length (i.e., including 5′ and 3′UTR sequences [56]) cells were seeded in DMEM medium onto 12-well plates using 2 × 10^5^ cells per well. After 24 h, cells were transfected with 500 ng of miRNA-expressing plasmid or 100 ng of sponge-expressing plasmid. Cells were incubated for 48 h, and subsequently RNA was extracted for gene expression quantification.

Total RNA isolated from MCF-7 cells was subjected to transcriptome sequencing (RNA-Seq; performed by Warsaw Genomics, Warsaw, Poland).

### 4.7. Protein Quantification

In parallel to transfections described above, cells were seeded onto 6-well plates and transfected using microRNA- or sponge-expressing plasmids using 2-fold higher amounts of all the reagents. Subsequently, cells were detached using Accutase (Sigma-Aldrich, Saint Louis, MO, USA) and protein was isolated using RIPA Lysis and Extraction Buffer (Thermo Fisher Scientific, Waltham, MA, USA) with 0.5 mM PMSF and cOmplete^TM^ protease inhibitor cocktail (Roche Diagnostics). Protein concentration was measured using Pierce BCA Protein Assay Kit (Thermo Fisher Scientific). Then, 2 μg of total protein was subjected to NIS quantification using Human NIS ELISA kit (ELK Biotechnology, Wuhan, China, cat. no. ELK4300), according to manufacturer’s instruction.

### 4.8. Radioactive Iodine Uptake Assay

The effect of miR-181a-5p on the metabolism of radioactive iodine (RAI) was analyzed in MCF-7 and HEK293-flhNIS, treated as described above. Then, 48 h after transfection, 10 µCi of Na^131^I (Polatom, Otwock, Poland) in a final concentration of 25 uM was added to each well. Plates were incubated for 30 min, washed with PBS twice and fresh medium was added. Retained activity was checked at Cyclone Plus (PerkinElmer, Shelton, CT, USA) by 10-min irradiation of phosphor imager screen. Results were normalized as percentage of activity of control-transfected cells.

### 4.9. Statistical Analysis

Each experimental series was compared with an appropriate control set. Normally distributed data were analyzed using the Student’s *t*-test, non-normally distributed data were analyzed using Wilcoxon and Mann–Whitney U tests and correlation analysis was performed using Spearman’s rank correlation coefficient (r). Statistical analysis was performed using GraphPad Prism [57]. *p*-values < 0.05 were considered significant.

## Figures and Tables

**Table 1:** Characteristic of PTC patients.

Feature		N	(%)
Sex	Female	43	88%
	Male	6	12%
Histopathological subtype	PTC cf	44	90%
	PTC fv	5	10%
No. of foci	Single	37	76%
	Multiple	12	24%
Tumor diameter	Average	16.23	mm
	Range	1–73	mm
pT feature	pT1a	21	43%
	pT1b	13	27%
	pT2	6	12%
	pT3	9	18%
	pT4	0	0%
pN feature	N0	39	80%
	N1a	5	10%
	N1b	5	10%
cM feature	M0	48	98%
	M1	1	2%
Vascular invasion	No	44	90%
	Yes	5	10%
Local invasion	No	33	67%
	Capsule only	10	20%
	Extrathyroidal	6	12%
Stage	I	39	80%
	II	2	4%
	III	5	10%
	IVA	2	4%
	IVB	0	0%
	IVC	1	2%
BRAF status ^1^	T (wild type)	23	56%
	T/A (mutated)	18	44%

^1^ BRAF^V600F^ mutation was checked in 41 out of 49 patients.

## Data Availability

The data presented in this study are available on request from the corresponding author.

## Supplementary Materials

The following are available online at https://www.mdpi.com/article/10.3390/ijms22116067/s1.
